# The long-term anterior segment configuration after pediatric cataract surgery and the association with secondary glaucoma

**DOI:** 10.1038/srep43015

**Published:** 2017-02-21

**Authors:** Ding Chen, Xian-hui Gong, He Xie, Xue-ning Zhu, Jin Li, Yun-e Zhao

**Affiliations:** 1 Eye Hospital, Wenzhou Medical University, Wenzhou, China

## Abstract

Secondary glaucoma constitutes major sight-threatening complication of pediatric cataract surgery, yet the etiology remains unclear. The purpose of this study was to investigate the long-term anterior segment configuration and the association with secondary glaucoma in pediatric pseudophakia. Ultrasound biomicroscopy (UBM) was performed on 40 eyes of 26 children underwent pediatric cataract surgery and intraocular lens (IOL) implantation. The anterior chamber depth (ACD), angle-opening distance at 500 μm (AOD500), trabecular-iris angle (TIA), central corneal thickness (CCT), structural abnormities, IOL position, IOP, and incidence of glaucoma were evaluated. High insertion of iris, in which the iris root is attached more anteriorly than normal, was seen in 13 eyes (32.50%). IOL was located in the capsular bag in 19 eyes and in the ciliary sulcus in 21 eyes. Logistic regression analysis identified high insertion of iris (OR 3.40, 95% CI 1.03–11.17, *p* = 0.03) and IOL implantation in sulcus (OR 1.39, 95% CI 1.07–4.85, *p* = 0.04) as independent risk factors for glaucoma. The presence of high insertion of iris and IOL implantation in ciliary sulcus may increase the long-term risk of the development of secondary glaucoma after pediatric cataract surgery.

Pediatric cataracts are a major cause of childhood blindness affecting approximately 200,000 children worldwide, with an estimated prevalence ranging from three to six per 10,000 live births^1^,^2^,^3^. The advances in surgical techniques and the development of intraocular lens (IOL) have considerably improved the outcome of pediatric cataract surgery over the past several decades. However, postoperative complications such as secondary glaucoma, posterior-capsule opacification (PCO), amblyopia, and axial growth are still major obstacles to long-term visual outcomes^4^,^5^,^6^,^7^. Secondary glaucoma with an incidence of 8.8–17% remains the most sight-threatening complication^4^,^8^,^9^,^10^,^11^,^12^. Unfortunately, the underlying etiology of secondary glaucoma in most cases remains unknown.

Unlike the adult eye, a child’s eye is still developing, and cataract surgery may significantly alter the normal structure of the anterior segment. This necessitates long-term observation of the anterior segment to assess the potential risk factors associated with the postoperative complications. Ultrasound biomicroscopy (UBM) has the advantage of revealing structures and anomalies that cannot be observed by optical devices, such as iridociliary tissue, lens opacification and posterior synechiae^13^. UBM has been performed on children to assess anterior segment developmental, such as persistent fetal vasculature (PFV) and posterior capsular support while considering secondary IOL implantation^14^,^15^. However, the data published is limited regarding the use of UBM for the assessment of the long-term configuration of pediatric pseudophakia and the possible correlation with secondary glaucoma.

In this study, we performed ultrasound biomicroscopy in patients who underwent pediatric cataract surgeries and IOL implantation with a long-term follow-up of at least five years. The UBM configuration of the anterior segment was analyzed both qualitatively and quantitatively. Our overall purpose was to uncover potential risk factors in respect to morphology that may be associated with secondary glaucoma after pediatric cataract surgery.

## Results

A total of 40 eyes of 26 patients eligible for inclusion criterion were enrolled in this study. Among them, 23 eyes of 14 children were diagnosed with congenital cataract and 17 eyes of 12 children were diagnosed with acquired cataracts. The detailed demographic and clinical characteristics of the patients who underwent pediatric cataract surgeries are summarized in Table 1. The mean follow up time was 7.98 ± 2.30 years (range, 5–11 years).

The median postoperative BCVA at the last visit was 0.40 (range 0.01–1.20). Overall, 17 eyes (42.5%) attained a BCVA of 0.5 or better. The mean refractive error (SE) was −1.39 ± 2.12 D (range −5.50– +4.63 D). Nystagmus was present in five patients, and strabismus was present in six patients, in which four were exotropia and two were esotropia. Posterior capsular opacification (PCO) was observed in eight eyes (20.00%). Nd-YAG laser capsulotomy for PCO was performed in seven eyes during the follow up, and surgical membranectomy was performed to clear the visual axis in one eye.

The mean IOP at the last visit was 17.29 ± 5.42 mm Hg (range, 10.2–31.4 mm Hg), which was significantly higher than the normal control (13.88 ± 3.52 mm Hg, range, 9.23–19.12 mm Hg) (*p* = 0.011). Glaucoma was diagnosed in nine eyes (22.5%) during the follow-up, among which, two eyes were diagnosed with angle-closure glaucoma (ACG) and seven eyes were diagnosed with open-angle glaucoma (OAG). One eye had borderline IOP without disc cupping and was diagnosed with suspected glaucoma case. These characteristics of glaucoma are listed in Table 2. The interval between cataract surgery and diagnosis of glaucoma was 4.42 ± 2.65 years (range 0.42–8.67 years). None of these was steroid-induced glaucoma. One eye with ACG underwent the removal of a pupillary membrane and peripheral iridectomy. Two eyes required surgical intervention with trabeculotomy. The remaining six eyes of glaucoma were treated with glaucoma medications alone.

The outcomes of ultrasound biometry and biomicroscopy of the anterior segment are listed in Table 3. There was no significant difference in CCT, ACD, AOD500, and TIA between pediatric pseudophakias and normal control. The mean CCT of glaucomatous pseudophakias was higher than that of non-glaucomatous pseudophakias (*p* = 0.04). The mean AOD500 of glaucomatous pseudophakias was significantly smaller than that of non-glaucomatous pseudophakias (*p* = 0.01). No significant difference was found in ACD and TIA between glaucomatous and non-glaucomatous pseudophakias (*p* = 0.22, 0.15, respectively).

**Figure 1:**
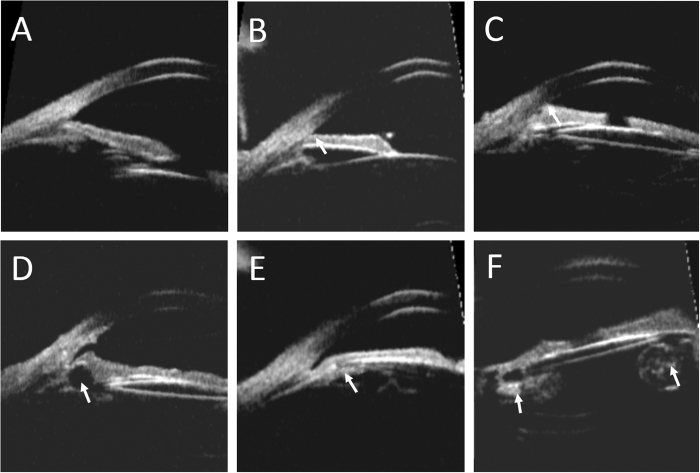
Abnormities in the configuration of the anterior segment in ultrasound biomicroscopy images. (**A**) The normal angle structure of a phakic normal control. (**B**) High insertion of iris in the pediatric pseudophakia. The iris root was located more anteriorly than that of normal control. (**C**) Peripheral anterior synechia of iris. (**D**) Image of an iridociliary cyst causing localized angle narrowing. (**E**) An enlarged Soemmering’s ring causing angle narrowing. (**F**) Image of residual lens material in the capsular bag.

High insertion of iris, in which the iris root was located more anteriorly than that of normal control (Fig. 1A,B), was observed in 13 eyes (32.50%) with pediatric pseudophakias. Peripheral anterior synechiae was seen in 5eyes (12.50%) (Fig. 1C). Most synechiae was localized and did not exceed two quadrants, especially close to the quadrants where the surgical incisions were located. The rate of high insertion of iris and peripheral anterior synechiae were higher in glaucomatous pseudophakia than in non-glaucomatous pseudophakia (*p* = 0.02, 0.015, respectively). Posterior synechiae to IOL was observed in two eyes (5.00%). Iridociliary cyst was seen in six eyes (15.00%), four were located in the ciliary body and two in the iris stroma. Large and superficial cysts led to localized angle narrowing (Fig. 1D). Enlarged Soemmering’s ring was observed in two eyes (5.00%), one of which showed general angle narrowing with borderline IOP (Fig. 1E). Residual lens material in the capsular bag was found in two eyes (5.00%) (Fig. 1F).

**Figure 2:**
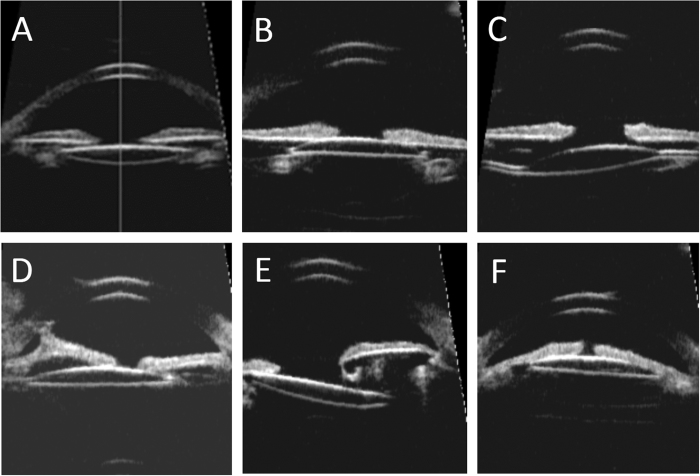
The intraocular lens (IOL) position in ultrasound biomicroscopy images. (**A**) IOL implantation in the capsular bag with good position. (**B**) IOL implantation in the ciliary sulcus with good position. (**C**) IOL tilting due to asymmetric fixation with optic and one haptic in the bag while the other haptic in the sulcus. (**D**) IOL decentration and anterior synechia of iris. (**E**) IOL subluxation due to insufficient support of capsular bag. (**F**) IOL forward shifting and embedding into iridociliary tissue.

IOL position was clearly observed and assessed by UBM (Fig. 2). Overall, 19 IOLs were located in the capsular bag (Fig. 2A) and 21 IOLs were located in the ciliary sulcus (Fig. 2B). The rate of IOL position (capsular bag vs. ciliary sulcus) was significantly different between glaucomatous pseudophakias and non-glaucomatous pseudophakias (*p* = 0.01). IOL malposition was observed in 11 eyes (27.50%). Two IOLs were tilting due to asymmetric fixation with optic and one haptic in the bag while the other haptic was located in the sulcus (Fig. 2C). IOL decentration was observed in seven eyes (58.33%) (Fig. 2D). IOL subluxation was observed in one eye due to insufficient support of the residual capsule (Fig. 2E). IOL forward shifting and embedding into iridociliary tissue was observed in one of the ACG eyes (Fig. 2F). Eleven eyes with IOL malposition were associated with IOL implantation in ciliary sulcus. There was no significant difference in the rates of IOL malposition between glaucomatous pseudophakias and non-glaucomatous pseudophakias.

Logistic regression model was used to reveal the potential morphological risk factors for secondary glaucoma, including covariates of AOD500, TIA, ACD, CCT, high insertion of iris, peripheral anterior synechia, and IOL implantation in sulcus (Table 4). In univariate analysis high insertion of iris, peripheral anterior synechia, and IOL implantation in sulcus were significantly associated with higher incidence of secondary glaucoma. Multivariate analysis identified high insertion of iris (OR 3.40, 95% CI 1.03–11.17, *p* = 0.03) and IOL implantation in sulcus (OR 1.39, 95% CI 1.07–4.85, *p* = 0.04) as independent risk factors for secondary glaucoma.

## Discussion

Secondary glaucoma is one of the most sight-threatening postoperative complications after pediatric cataract surgery^10^,^16^. The reported long-term incidence of secondary glaucoma varies, ranging from 10–17%^4^,^8^,^9^,^10^,^11^. The mean interval between cataract surgery and diagnosis of glaucoma has been reported to be 4.0–5.2 years^8^,^17^,^18^. The incidence tends to be higher if patients are followed up for longer periods. The Infant Aphakia Treatment Study (IATS) reported an incidence of 8.8% of glaucoma at the one-year follow-up visit, while the incidence of glaucoma of the same cohort increased to 17% according to the five-year study^10^,^12^. In our study, nine eyes (22.50%) were diagnosed with secondary glaucoma and had an average follow up of more than five years. This slightly higher incidence than the previous reports is probably due to the longer follow-up time in our study.

Numerous risk factors for the development of secondary glaucoma have been noted, including younger age at detection of cataract, small corneal diameters, coexistence of PFV, a family history of aphakic glaucoma, surgery in the first year of life, and primary posterior capsulotomy/anterior vitrectomy performed at the time of cataract surgery^11^,^12^,^19^,^20^. However, the underlying etiology of secondary glaucoma in most cases remains unknown. Glaucoma may occur because of the abnormalities and disorganization of the ocular structure caused by pediatric cataract surgery. Few studies have focused on the anatomical characteristics of the pediatric pesudophakia. In this study, we investigated the long-term anterior segment configuration after pediatric cataract surgery with ultrasound biomicroscopy, and analyzed the potential risk factors in morphological aspect that may be associated with secondary glaucoma.

The mean angle-opening distance and trabecular-iris angle in pediatric pseudophakias were smaller in value than phakia in normal controls, but the difference was not significantly different. This finding is slightly different from the study conducted by Nishijima *et al*.^15^ They measured the angle structure with UBM in 15 children after congenital cataract surgery and found out that the angle-opening distance, but not the trabecular-iris angle, was significantly less than normal controls. However, similar findings were observed in the glaucomatous pseudophakias vs. non-glaucomatous pseudophakias in our study. Nishijima*et al*.^15^ speculated that the scleral spur was located more posteriorly in eyes with congenital cataracts than normal eyes. They reported that 75.0% eyes with congenital cataracts had that type of angle configuration abnormality and linked it to an inclination of elevation in IOP after surgery. Walton^21^ conducted gonioscopy in 65 patients with aphakic secondary glaucoma and found a circumferential repositioning of the iris insertion anteriorly at the level of the posterior or midtrabecular meshwork, with resultant loss to view of the ciliary body band and scleral spur in 79 eyes of open-angle glaucoma. We attribute this angle abnormality to the more anteriorly attached iris root rather than more posteriorly located scleral spur comparing to the normal phakia, as claimed by Nishijima^15^. In our study, this abnormality, termed high insertion of iris, was frequently observed in nearly 1/3 of pediatric pseudophakias and in more than half of glaucomatous pseudophakias. Logistic regression analysis showed the presence of high insertion of iris increased the risk for developing glaucoma by 3.40 times. This suggests that high insertion of iris may be an important independent risk factor for the secondary glaucoma after pediatric cataract surgery.

Compared to open-angle glaucoma, the etiology for angle-closure glaucoma is more definitive. Angle-closure glaucoma may occur early after pediatric cataract surgery, probably due to the heavy postoperative inflammatory response leading to synechia formation in the chamber angle, or pupillary block and iris bombé^16^. Peripheral anterior synechia was obsevered by UBM in five eyes in our study. Most synechiae were localized and located near the quadrants where surgical incisions were made. In the two eyes that had secondary angle-closure glaucoma after surgery, signs of peripheral anterior synechia was observed in more than three quadrants. Posterior synechiae along with forward shifting of IOL and anterior chamber shallowing was also seen in one eye. These old signs of synechiae and pupillary block do not seem to regress over time, even after glaucoma treatment, indicating the underlying etiology of this type of glaucoma.

Other configurational abnormities that may potentially compromise the angle structure were revealed by UBM. Iridociliary cysts were not uncommon in pediatric pseudophakia in our cases (six eyes, 15%). Katsimpris *et al*.^22^ reported a single case of chronic angle-closure glaucoma caused by multilobulate iridociliary cysts. In our study, cysts were mostly small and individual, and only large and superficial cysts led to localized angle narrowing, but none caused angle-closure glaucoma. More extensive chronic angle narrowing is probably due to the enlarging Soemmering’s ring. Kung^23^ and more recently Kitamura^24^ reported cases of angle-closure glaucoma in adult pseudophakias caused by an enlarged Soemmering’s ring. We observed two eyes with enlarged Soemmering’s rings leading to a small angle-opening distance and trabecular-iris angle in all quadrants, and one eye had an elevated borderline IOP. Nihalani^25^ found that the incidence of glaucoma might be higher in secondary IOL implantation in pediatric aphakia without debulking the Soemmering’s ring. Residual lens material does not seem to affect the angle directly, which was seen in two eyes in our study. However, in rare circumstances, lens-induced glaucoma can occur long after pediatric cataract surgery^26^. Therefore, the configurational abnormities, mentioned above, may warrant long-term follow-up. In addition to the abnormities in angle structure, the IOL position may also be associated with the incidence of secondary glaucoma. In our study, seven of 9 eyes with secondary glaucoma had IOLs implanted in ciliary sulcus. Logistic regression analysis suggested that IOL implantation in sulcus increased the risk for developing glaucoma by 1.39 times. Nihalani^25^ found that the incidence of glaucoma was higher in patients with a secondary IOL implantation in sulcus compared to implantation in the capsular bag. Asrani^27^ reported that implantation of an IOL into the capsular bag may inhibit the development of secondary glaucoma. Although the advantage of in-capsular implantation of IOL is unambiguous, the major challenge in secondary IOL implantation in pediatric aphakia is the difficulty of opening the capsular bag and clearing the Soemmering’s ring. In our study, most eyes that underwent secondary IOL implantation resulted in IOL placement in ciliary sulcus. IOL implantation in sulcus may increase the risk of iris chaffing, posterior synechia, and chronic uveal inflammation^28^, however, the underlying mechanism of increased risk of secondary glaucoma remains undetermined. In addition, IOL implantation in sulcus increases the chance of IOL malposition. Eleven of 12 eyes with IOL malposition were associated with implantation in ciliary sulcus. Nonetheless, except for the single case of angle-closure glaucoma showing the forward shifting of IOL due to the early event of posterior synechia and pupillary block, there is no direct evidence in our study suggesting that IOL malposition alone is associated with secondary glaucoma.

The aim of pediatric cataract treatment is to achieve good visual function for life. Beside secondary glaucoma, there are multiple factors that contribute to the visual outcome of pediatric cataract surgery, such as type of cataract, age at surgery, surgical technique, postsurgical refractive correction, compliance of amblyopia therapy, and other postoperative complications^4^,^5^,^6^,^7^,^29^,^30^,^31^. Fortunately, the visual outcomes of most pediatric cataract surgery are satisfactory with the proper postoperative care and amblyopia therapy. In our study, the median of postoperative BCVA was 0.40, and 17 eyes (42.5%) obtained a BCVA of 0.5 or better after more than five years of follow-up. These results are comparable to other published visual outcomes of children undergoing surgical treatment for pediatric cataracts^29^,^30^,^31^.

The limitations of this study include the retrospective design and the relatively small sample size. In addition, the influence of angle structure and IOL position on the incidence of secondary glaucoma may be biased by the age at surgery. Nevertheless, surgery performed by the same experienced surgeon, long-term follow-up for more than five years, and detailed ultrasound biomicroscopic analysis of the anterior segment in pediatric pseudophakia are major strengths of this study.

In conclusion, the anterior segment configuration of the pediatric pseudophakias is significantly altered by cataract surgery and the structural abnormities are not uncommon revealed by ultrasound biomicroscopy. The high insertion of iris and IOL implantation in ciliary sulcus may significantly increase the long-term risk of secondary glaucoma after surgery. Periodical ultrasound biomicroscopy is recommended for the evaluation of the anterior segment during the follow-up of pediatric cataract surgery.

### Patients and methods

This was a retrospective, observational case series. Children diagnosed with congenital/acquired cataracts underwent cataract surgery and intraocular lens (IOL) implantation in the Eye Hospital of Wenzhou Medical University from January 2005 to December 2010. Patients that were followed for at least five years were included in this study. The medical records of all patients were reviewed. Eyes with microcornea, whose horizontal diameter at the time of cataract surgery was less than 9.0 mm, and with primary disease (e.g., chronic anterior uveitis, trauma, anterior segment dysgenesis, optic nerve or other fundus abnormalities, prematurity and cataract associated with other syndromes, maternal rubella syndrome and systemic disorders) were excluded. Patients with signs of congenital glaucoma before surgery, including increased intraocular pressure (IOP) along with increased corneal diameter and corneal edema, were also excluded. Twenty four eyes of 12 age-matched healthy phakic subjects were enrolled as control subjects.

Ethical approval for this study was provided by the Ethical Committee of Wenzhou Medical University. All measurements followed the tenets of the Declaration of Helsinki, and writen informed consents were obtained from the legal guardians of the minor subjects enrolled in the study.

#### Surgical technique

All surgeries were performed by the same experienced surgeon (Y.E.Z.) under general anesthesia. Variation in surgical technique occurred in some patients due to the age of the patient and when the surgery was performed. Patients younger than 6 months were treated with a pars plana or limbal lensectomy and anterior vitrectomy, while the remaining patients were treated with phaco-aspiration with or without primary posterior capsulotomy and anterior vitrectomy. All implanted IOLs were Acrysof IOLs (Models SA60AT, SN60AT, MA60AC, and MA60BM) (Alcon surgical, Fort Worth, Texas, USA). Patients underwent primary IOL implantation at the time of lens removal or postponed secondary IOL implantation after a period of spectacle use after lens removal, depending on the age and situation of the patient at time of surgery. IOL was placed in the ciliary sulcus in cases of intraoperative posterior capsule defect or rupture, or failing to open the capsular bag during the secondary IOL implantation.

The operated eyes were treated with topical antibiotics, corticosteroids, NSAID, and mydriatic and cycloplegic agents after surgery. Prednisolone acetate 1% was initially used 4 times a day for the first week, and then tapered down over 1 month to lower the incidence of steroid-induced ocular hypertension. The use of eye patches, ranging from three to six hours per day, was prescribed immediately after surgery to manage amblyopia.

#### Postoperative follow-up and ophthalmological examinations

Patients were followed up at postoperative 1 week, 1 month, 3 months, and then every 6 months at our hospital. The best-corrected visual acuity (BCVA) was assessed using a Snellen chart with the patient wearing optimal refractive correction. The power of corrective lenses was determined by retinoscopy. The refractive error was calculated as the spherical equivalent (SE). Strabismus was examined with the Krimsky test. IOP was evaluated with a Perkins handheld applanation tonometer under anesthesia. Indirect ophthalmoscopy was used to examine the fundus after dilation. All postoperative complications were recorded. Secondary glaucoma following cataract surgery was diagnosed according to the criteria established by the Infant Aphakia Treatment Study^12^.

#### Ultrasound biomicroscopy of the ocular anterior segment

Ultrasound biomicroscopy examination was performed for all subjects using the OTIScan 2000 (Optos Hialeah, Hialeah, FL, USA). Scanning was conducted with the nondilated eye in a central position and the patient in the supine position. A drop of a topical anesthetic agent (benoxinate hydrochloride) was applied to both eyes. A cup suitable to the patient’s palpebral fissure size was then inserted between the lids. Sterile normal saline solution was used to fill the cup to the appropriate level. The UBM probe with the 35 MHz tip was used to obtain two radial scans at the 12 o’clock and 3 o’clock positions (from the far anterior to the anterior corneal surface to the posterior to the posterior lens capsule) and remained perpendicular to the ocular surface and central in the pupil. Scan reviews were performed at a later time. Clips of the scans of all eyes were reviewed. The best quality frames that were most illustrative were chosen for data acquisition. In addition, central corneal thickness (CCT) was obtained using the contact method with the A-scan biometry probe, included in the OTIScan2000.

**Figure 3:**
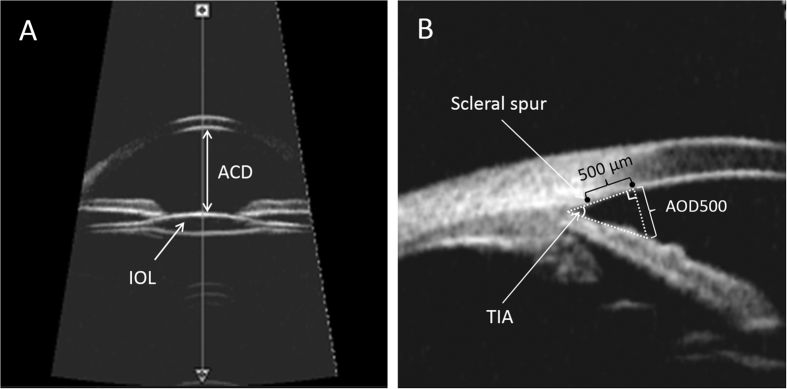
Ultrasound biomicroscopy (UBM) of the anterior segment of pediatric pseudophakia. (**A**) UBM images of the anterior chamber and intraocular lens (IOL). Anterior chamber depth (ACD) was determined from the central inner corneal surface, perpendicular to the corneal surface to the most anteriorly visible part of the IOL. (**B**) Quantitative angle measurement. Angle-opening distance at 500 μm (AOD500) was measured on a line perpendicular to the trabecular meshwork at points 500 μm from the scleral spur. Trabecular-iris angle (TIA) was measured with the apex in the iris recess and the arms of the angle passing through a point on the trabecular meshwork 500 μm from the scleral spur and the point on the iris perpendicularly opposite.

Anterior chamber depth (ACD) was determined from the central inner corneal surface, perpendicular to the corneal surface to the most anteriorly visible part of the IOL (Fig. 3A). The angle were evaluated by using the angle-opening distance at 500 μm (AOD500) and the trabecular-iris angle (TIA), as proposed by Pavlin and associates^13^. AOD500 was measured on a line perpendicular to the trabecular meshwork at points 500 μm from the scleral spur using calipers of the software in a high-magnification view, and TIA was measured with the apex in the iris recess and the arms of the angle passing through a point on the trabecular meshwork 500 μm from the scleral spur and the point on the iris perpendicularly opposite (Fig. 3B). All four-quadrant values were obtained and averaged for statistical analysis. Any abnormities in the configuration of the anterior segment including the angle, iris, ciliary body, or capsule was described and documented (Fig. 1). The IOL position was also analyzed base on the four-quadrant ultrasound biomicroscopic images (Fig. 2).

#### Data analysis

Statistical analyses were performed using software SPSS version 17.0 (SPSS Inc., Chicago, IL, USA). All data was analyzed for normality using the Kolmogorov–Smirnov test, which indicated several instances of non-normal distributions and recommended non-parametric statistical analyses. Values were expressed as mean ± SD (range) or median (range). Independent sample t-test or Mann–Whitney test was employed to compare parameters between different groups. Chi-Square test or Fisher’s exact test was used to compare the rates. Univariable and multivariable logistic regression models were used to assess the influence of a selected set of morphological characteristics of the anterior segment on the incidence of secondary glaucoma. Odds ratios (OR) with 95% confidence intervals (CI) were reported. A *p*-value < .05 was considered statistically significant.

## Additional Information

**How to cite this article**: Chen, D. *et al*. The long-term anterior segment configuration after pediatric cataract surgery and the association with secondary glaucoma. *Sci. Rep.* **7**, 43015; doi: 10.1038/srep43015 (2017).

**Publisher's note:** Springer Nature remains neutral with regard to jurisdictional claims in published maps and institutional affiliations.

## Figures and Tables

**Table 1:** Demographics and clinical characteristics of children that underwent pediatric cataract surgeries.

Parameter	Value
No. of eyes/patients	40/26
Sex, No. (%) of Female	12 (46.15%)
Laterality, No. (%) of OD	24 (60.00%)
Age at primary surgery, years (range)	3.06 ± 3.70 (0.17–9.25)
Primary vs Secondary IOL implantation, eyes	17/23
Interval between primary surgery and secondary IOL implantation, years (range)	2.53 ± 1.25 (0.50–5.00)
Age at last visit, years (range)	11.12 ± 2.48 (7.83–15.17)
Anterior segment vitrectomy, No. (%) of eyes	29 (72.50%)
Posterior capsulectomy, No. (%) of eyes	34 (85.00%)
Peripheral iridotomy, No. (%) of eyes	6 (15.00%)
Preoperative IOP, mm Hg (range)	10.51 ± 2.08 (7.19–14.52)

IOP = intraocular pressure; IOL = Intraocular lens.

**Table 2:** Characteristics of eyes diagnosed with secondary glaucoma following pediatric cataract surgery.

No.	Laterality	Glaucoma type	Interval between diagnosis and initial surgery (years)	IOP at last visit	Treatment	CCT (μm)	Ultrasound biomicroscopic features			
							ACD (mm)	AOD500 (μm)	TIA (degrees)	Abnormities in configuration
1	od	ACG	1.50	14.86	surgery	563	2.90	402	32.98	PAS
2	os	ACG	0.42	27.12	surgery	572	2.58	395	29.62	PAS/posterior synechiae
3	os	OAG	3.25	18.98	medication	533	3.46	389	46.93	High insertion of iris
4	od	OAG	7.00	19.94	medication	541	4.14	371	43.78	High insertion of iris
5	od	OAG	3.17	20.27	medication	576	3.51	465	52.34	—
6	od	OAG	5.00	23.64	medication	587	4.08	403	47.52	High insertion of iris
7	od	OAG	4.33	19.27	surgery	543	3.69	468	50.11	—
8	os	OAG	6.42	31.40	medication	622	3.90	383	42.10	High insertion of iris
9	os	OAG	8.67	25.52	medication	605	3.97	437	45.41	High insertion of iris

ACG = angle-closure glaucoma; OAG = open-angle glaucoma; IOP = intraocular pressure; CCT = central corneal thickness; ACD = Anterior chamber depth; AOD500 = angle-opening distance at 500 μm; TIA = trabecular-iris angle; PAS = peripheral anterior synechia.

**Table 3:** Outcomes of ultrasound biometry and biomicroscopy of the anterior segment in pediatric pseudophakia and normal controls.

	Paediatric pseudophakia (n = 40)	Normal controls (n = 24)	Glaucomatous pseudophakia (n = 9)	Non-glaucomatous pseudophakia (n = 31)
CCT (μm)	560.29 ± 47.60 (496–622)	552.89 ± 38.61 (507–598)	571.33 ± 30.06 (533–622)**	557.11 ± 41.32 (496–603)
ACD (mm)	3.65 ± 0.67 (2.58–4.52)	3.78 ± 0.46 (3.24–4.37)	3.57 ± 0.53 (2.58–4.14)	3.67 ± 0.59 (2.90–4.52)
AOD500 (μm)	447.52 ± 68.15 (371–528)	463.42 ± 57.71 (408–535)	412.55 ± 35.51 (371–468)**	459.23 ± 67.61 (359–528)
TIA (degrees)	46.31 ± 9.41 (29.62–59.49)	51.86 ± 8.27 (35.98–61.50)	43.42 ± 7.57 (29.62–52.34)	47.14 ± 9.02 (34.71–59.49)
High insertion of iris (No. (%))	13 (32.50%)*	0 (0%)	5 (55.56%)**	8 (25.81%)
Peripheral anterior synechiae (No. (%))	5 (12.50%)*	0 (0%)	2 (22.22%)**	3 (9.68%)
Posterior synechiae (No. (%))	2 (5.00%)*	0 (0%)	1 (11.11%)	1 (3.23%)
Iridociliary cysts (No. (%))	6 (15.00%)*	1 (4.17%)	1 (11.11%)	5 (16.13%)
Enlarged Soemmering’s ring (No. (%))	2 (5.00%)	NA	0 (0%)	2 (6.45%)
Residual lens material (No. (%))	2 (5.00%)	NA	0 (0%)	2 (6.45%)
IOL position: capsular bag vs ciliary sulcus (No.)	19/21	NA	2/7**	17/14
IOL malposition (No. (%))	11 (27.50%)	NA	3 (33.33%)	8 (25.81%)

CCT = central corneal thickness; ACD = Anterior chamber depth; AOD500 = angle-opening distance at 500 μm; TIA = trabecular-iris angle; IOL = introcular lens, peripheral anterior synechiae; NA = not applicable. *For significant difference in parameters between pediatric pseudophakia and normal controls; **For significant difference in parameters between glaucomatous pseudophakia and non-glaucomatous pseudophakia.

**Table 4:** Univariable and multivariable logistic regression investigating the association between morphological predictors and detection of secondary glaucoma.

Predictors	Univariate Analysis			Multivariate Analysis		
	OR	95% CI	P	OR	95% CI	P
AOD500 (μm)	0.34	0.11–1.85	0.43	0.22	0.09–1.77	0.12
TIA (degrees)	0.97	0.87–1.09	0.62	1.02	0.88–1.17	0.82
ACD (mm)	0.63	0.11–3.60	0.60	0.34	0.28–2.83	0.33
CCT	0.99	0.71–1.05	0.09	0.95	0.69–1.12	0.12
High insertion of iris	3.97	1.31–10.75	0.01	3.40	1.03–11.17	0.03
Peripheral anterior synechiae	1.34	1.03–4.76	0.04	1.26	0.72–3.99	0.10
IOL implantation in sulcus	1.75	1.18–5.26	0.02	1.39	1.07–4.85	0.04

AOD500 = angle-opening distance at 500 μm; TIA = trabecular-iris angle; ACD = Anterior chamber depth; CCT = central corneal thickness; IOL = introcular lens; OR = odds ratio; CI = 95% confidence intervals.
